# Real‐World Treatment Patterns and Outcomes of Zanubrutinib in Chronic Lymphocytic Leukemia and Small Lymphocytic Leukemia (CLL/SLL)

**DOI:** 10.1002/cam4.71300

**Published:** 2025-10-15

**Authors:** Margaret Li Krackeler, Bryant H. Chee, Arthur K. Orchanian, Raymond Liu, Lisa Law, Shiyun Zhu, Zheng Zhu, Lori C. Sakoda, Susan Buchanan, Marjan Massoudi, Alfredo Lopez, Jahan Tavakoli

**Affiliations:** ^1^ Department of Hematology Oncology Kaiser Permanente San Francisco San Francisco California USA; ^2^ Department of Internal Medicine Kaiser Permanente San Francisco San Francisco California USA; ^3^ Department of Hematology Oncology Kaiser Permanente Roseville Roseville California USA; ^4^ Division of Research Kaiser Permanente Northern California Pleasanton California USA; ^5^ Department of Health Systems Science Kaiser Permanente Bernard J. Tyson School of Medicine Pasadena California USA; ^6^ BeOne Medicines Ltd San Carlos California USA

**Keywords:** chronic lymphocytic leukemia, ibrutinib, real‐world data, zanubrutinib

## Abstract

Zanubrutinib is a next‐generation selective Bruton's tyrosine kinase inhibitor (BTKi) with superior efficacy and lower cardiotoxicity rates seen in clinical trials over first‐generation ibrutinib in CLL/SLL patients. We present real‐world treatment patterns based on a practice change from ibrutinib to zanubrutinib in CLL/SLL patients in an integrated community oncology practice. We retrospectively analyzed adult patients who filled at least 3 months of zanubrutinib at Kaiser Permanente Northern California. Treatment patterns, adverse events (AE), and mortality were reported. Two hundred eighty‐one patients received zanubrutinib; 190 patients switched from ibrutinib. Similar AE rates were seen with both BTKis, with a lower proportion of patients with treatment‐limiting AEs and lower cardiac AE rates with zanubrutinib. Most patients remained on zanubrutinib at the end of data collection without treatment‐related deaths reported. In the real‐world setting, zanubrutinib is effective and safe in patients with or without prior ibrutinib use, with low cardiotoxicity and AE rates, though data was limited by a difference in follow‐up time.

Dear Editor,

1

Zanubrutinib is a next‐generation selective covalent Bruton's tyrosine kinase inhibitor (BTKi) with higher BTK specificity and tolerability than first‐generation BTKi [1]. Zanubrutinib significantly improved progression‐free survival (PFS) in both the treatment‐naive setting when compared to chemoimmunotherapy in the SEQUOIA trial and the subsequent line setting compared to ibrutinib in the ALPINE trial with fewer adverse events (AEs) [2, 3]. The initial results of the ALPINE trial presented at the European Hematology Association and American Society of Clinical Oncology in 2021 led to a practice change at Kaiser Permanente Northern California (KPNC) to convert ibrutinib to zanubrutinib as the preferred BTKi for CLL/SLL in August 2021 [4, 5]. Zanubrutinib was FDA approved in January 2023 based on updated ALPINE data presented at the American Society of Hematology in 2022, and extended 3‐year follow‐up data for both trials maintained superior PFS benefit [6, 7, 8]. Here we present real‐world treatment patterns in treatment‐naïve, BTKi‐naïve, and ibrutinib‐treated CLL/SLL patients after a practice change to zanubrutinib in an integrated community oncology practice.

We retrospectively analyzed adult CLL/SLL patients 18 years and older who filled at least 3 months of zanubrutinib therapy from October 1, 2018, to September 15, 2023, at KPNC identified by pharmacy medication fill reports. Patients were excluded if they participated in a clinical trial or did not have health plan membership within 6 months of the first fill. A 3 month duration of fills was chosen to allow for sufficient data and follow‐up for zanubrutinib use in a real‐world setting. The data collection period was January 1, 2016, to December 31, 2023. In August 2021, pharmacy‐identified patients with CLL/SLL on ibrutinib and their providers worked to determine if a switch to zanubrutinib was appropriate based on emerging evidence. Treatment patterns including duration (defined as time between first and last medication fill), dose modifications and discontinuation rates, treatment‐emergent AEs (TEAE: any AE reported during BTKi use with or without dose modification), treatment‐limiting AEs (TLAE: any AE that led to BTKi discontinuation), and mortality with ibrutinib and zanubrutinib were ascertained via chart review of provider and pharmacy notes. Data extraction and analysis were performed with *SAS studio 3.81* and *R v4.3.1*. Study procedures were approved by the KPNC institutional review board under a waiver of informed consent.

In total, 281 patients received a minimum of 3 months' fill of zanubrutinib; 190 patients switched from ibrutinib (ibru‐zanu), and 91 patients received zanubrutinib only. Seventy‐five patients were excluded for < 3 months of fills, with 40 patients picking up their first fill. The median age was 71 years old. Most patients were male (64%), and of White (75%) followed by Black (10%) race. Compared with ibru‐zanu patients, zanubrutinib‐only patients were older (median age 74 vs. 69) but were comparable in sex, race, insurance type, and prior cardiac comorbidities (Table 1). High‐risk patients included 26 patients (9%) with del(17p), 14 patients (5%) with unmutated IGHV, and 13 patients (5%) with complex karyotype, though *TP53* mutation status was unavailable for the entire cohort. The primary reasons for switching to zanubrutinib were practice change (57%) and progression from prior CLL‐directed therapy (12%), with nine of those patients having reported progression on ibrutinib. This decision was made at the discretion of the treating provider, with eight of the nine patients still on zanubrutinib at the end of follow‐up, with one patient stopping due to AE. Sixty‐onepatients (22%) were treatment‐naïve prior to zanubrutinib initiation, with an additional 24 patients who were BTKi‐naïve. Of the 190 patients who switched from ibrutinib, 39% were treatment‐naïve prior to ibrutinib initiation. Median follow‐up time after initiation of the first BTKi was longer in the ibru‐zanu group, with a median duration of 45 months for ibrutinib therapy from initiation to switch as compared to 7.8 months in patients who received zanubrutinib as the first BTKi. Median follow‐up of zanubrutinib use in patients with prior ibrutinib treatment was 24.1 months (Table 2). Of note, the treatment period of ibrutinib was prior to 2021 when salvage options were limited. Five patients had < 3 months of ibrutinib therapy fill prior to zanubrutinib, with three patients stopping ibrutinib due to AE of headache, fatigue, and atrial fibrillation. Only one patient experienced a TEAE on zanubrutinib but continued therapy to the end of follow‐up (Table S1).

**TABLE 1:** Demographics.

	Overall (*N* = 281)	Ibru to Zanu (*n* = 190)	Zanu as 1st BTKi (*n* = 91)
Age at 1st BTKi fill, median (range)	71 (64, 76)	69 (62, 75)	74 (66, 80)
Sex, *n* (%)			
Male	181 (64)	117 (62)	64 (70)
Female	100 (36)	73 (38)	27 (30)
Race/ethnicity, *n* (%)			
White	210 (75)	138 (73)	72 (79)
Black	28 (10)	22 (12)	6 (7)
Other	43 (15)	30 (16)	12 (14)
Charlson Comorbidity Index, mean (SD)	2.1 (1.8)	2.0 (1.8)	2.4 (1.8)
Antiplatelet or anticoagulation use, *n* (%)	110 (39)	75 (39)	35 (38)
Prior cardiac comorbidities (AFib, CHF, CAD), *n* (%)	71 (25)	48 (25)	23 (25)
Insurance type, *n* (%)			
Commercial	87 (31)	60 (32)	27 (30)
Medicaid	7 (2.5)	7 (3.7)	0 (0)
Medicare	186 (66)	122 (64)	64 (70)
Treatment‐naïve, *n* (%)	61 (22)	0 (0)	61 (67)

Abbreviations: AFib, atrial fibrillation; BTKi, Bruton's tyrosine kinase inhibitor; CAD, coronary artery disease; CHF, congestive heart failure; Ibru, Ibruntinib; Zanu, Zanubrutinib.

**TABLE 2:** Outcomes of BTKi treatment.

	Ibrutinib	Zanubrutinib		
	Overall (*N* = 190)	Overall (*N* = 281)	Ibru‐Zanu switchers (*n* = 190)	Zanu only (*n* = 91)
Median follow‐up, mos. (range)	44.7^a^ (11.2, 114)	23.3^b^ (2.2, 25.9)	24.1^c^, ^d^ (2.2, 25.9)	7.8^c^ (2.2, 25.3)
Median treatment duration, mos. (range)^d^	20.7 (0.03, 88.4)	20.4 (1.8, 25.3)	22.8 (1.8, 25.3)	6.7 (1.9, 24.8)
TEAE, *n* (%)	69 (36.3)	88 (31.3)	56 (29.5)	32 (35.2)
TLAE, *n* (%)	21 (11.1)	22 (7.8)	14 (7.4)	8 (8.8)
CTCAE grade of TLAE ≥ 3, *n* (%)	7 (3.6)	4 (1.4)	3 (1.6)	1 (1.1)
Cardiotoxicity, *n* (%)				
EAIR (95% CI)	0.31 (0.29, 0.33)	0.16 (0.14, 0.18)	0.16 (0.14, 0.19)	0.07^e^ (0.00, 0.21)
TEAE	18 (9.5)	6 (2.1)	5 (2.6)	1 (1.1)
TLAE	8 (4.2)	2 (0.7)	2 (1.1)	0 (0)

Abbreviations: BTKi, Bruton's tyrosine kinase inhibitor; CTCAE, common terminology criteria for adverse events; EAIR, exposure‐adjusted incidence rate; ibru, ibrutinib; TEAE, treatment‐emergent adverse event; TLAE, treatment‐limiting adverse event; zanu, zanubrutinib.

^a^ All ibrutinib patients from ibru initiation to end of follow‐up (death or study end).

^b^ All zanubrutinib patients from switch if prior ibru use or zanu initiation if no prior ibru use to end of follow‐up.

^c^ Zanubrutinib use starting from zanu start to end of follow‐up.

^d^ Patients with < 3 months' fill of zanu were excluded; however, those who had more than 3 months of supply but discontinued treatment before exhausting the 3‐month supply were retained in the cohort.

^e^ Zanu only group was standardized to the age and sex distribution of Ibru‐Zanu switchers with age at first‐line BTKi using indirect standardization.

Similar TEAE percentages were seen with the use of both BTKi therapies, with lower TLAE incidence with zanubrutinib (Table 2). The most common TLAEs were atrial fibrillation (1.6%) and fatigue (1.6%) for ibrutinib, and cytopenia (1.4%) and rash/bruising (1%) for zanubrutinib. Five of 10 patients who reported any bruising—three on ibrutinib and two on zanubrutinib—had concurrent antiplatelet/anticoagulation use. Seventeen patients (6%) switched to zanubrutinib due to AE from prior treatment, 10 of which were from ibrutinib specifically. Of all 69 patients who reported TEAEs with ibrutinib use, 12 patients (17%) had recurrence of their prior ibrutinib AE with zanubrutinib use. Thirty‐eight patients (13%) received IVIG or viral (HBV or CMV) prophylaxis during BTKi treatment without any reported reactivation. Cardiotoxicity exposure‐adjusted incidence rates were higher with ibrutinib than with zanubrutinib (Table 2). Eleven patients developed atrial fibrillation on ibrutinib and fourpatients on zanubrutinib, leading to discontinuation in three and one patients, respectively. Dose modification occurred in 34 patients on ibrutinib and 45 patients on zanubrutinib primarily due to AE (11%) or patient request (6%).

Of the 281 patients who received zanubrutinib, 79% remain on treatment at follow‐up. Of the high‐risk patients, 15 of 17 patients in the zanubrutinib‐only group were still on zanubrutinib; two patients stopped for AE or own request at the end of follow‐up. Sixteen of 23 high‐risk patients in the ibru‐zanu group were still on zanubrutinib, while four patients progressed, and three patients stopped due to AE by the end of follow‐up. Of the nine patients with prior progression on ibrutinib, one discontinued due to recurrent skin infections on zanubrutinib. The other eight patients, including one who developed grade 1 atrial fibrillation, remained on zanubrutinib with continued response. Fifty‐nine patients (21%) discontinued zanubrutinib, with the reasons including: TLAE in 22 patients (7%) primarily for CTCAE grade 1–2 AE after patient and provider discussion, progression in 16 patients (6%), death in 12 patients (4%), patient request in 6 patients (2%), and nonadherence in 3 patients (1%). Additional mutational analysis was not routinely performed upon progression. During treatment, eight deaths occurred from infection, including five from COVID‐19.

In this cohort of patients in a real‐world community practice setting, zanubrutinib was effective for the treatment of CLL/SLL with an overall median follow‐up of 23.3 months. As seen in ALPINE and SEQUOIA trials, zanubrutinib was well tolerated, with low rates of any AE or cardiotoxicity while on therapy [2, 3, 4]. Analysis of patients who received zanubrutinib as their first BTKi was limited by the short duration of follow‐up but demonstrated low rates of all AEs and cardiotoxicity (Table 2). The shorter follow‐up for this cohort was expected as the median duration between CLL diagnosis and the first BTKi fill is approximately 3 years, longer than the time between practice change in August 2021 and last follow‐up in December 2023. In our study, as compared to the SEQUOIA and ALPINE clinical trials, low rates of infections, bleeding, and cardiotoxicity were seen. The most common TEAEs that occurred were cytopenia, fatigue, and myalgias. Consistent with current real‐world research, patients often discontinued due to CTCAE grade 1–2 AEs that are often underreported in clinical trials but significantly affect patients' experiences with therapy [9]. Particularly with CLL/SLL, a chronic disease often requiring lifelong therapy, long‐term observation with real‐world analysis of large patient cohorts is crucial in assessing patient preferences and outcomes to further enhance both patient and provider clinical decision making.

The rapid change in provider practice based on emerging data is unique in size and scale, and its implementation capability in KPNC is ideal for real‐world studies. KPNC is a network of 21 community cancer centers in an integrated healthcare system that services 4.5 million members that represent the general population [10]. Especially uncommon in oncology, we were able to systematically recommend a transition to an updated treatment that had shown improved efficacy and safety in a large cohort of patients, with the ultimate decision for any therapy change at the provider and patients' discretion. This allowed a unique perspective from which to analyze the real‐world differences between ibrutinib and zanubrutinib treatment patterns. Additionally, crossover data were analyzed to evaluate the efficacy and safety of next‐generation BTKi use after initial ibrutinib use.

Limitations of this study include incomplete genomic data, which affects high‐risk subgroup interpretation, differences in follow‐up time while on ibrutinib and zanubrutinib, and exclusion of patients with < 3 months of fills of zanubrutinib. Given the timing of practice change in August 2021, a shorter follow‐up time on zanubrutinib therapy was observed, which limited survival analysis. This difference in duration could account for findings of tolerability. Additionally, excluding patients with < 3 months of fills was done to ensure an adequate treatment duration for AE reporting but could have eliminated patients who had poor tolerability of zanubrutinib. Response rates were difficult to ascertain, and AEs could have been underreported due to limitations in real‐world electronic health record data and observational research. There was a possibility of bias regarding tolerability of zanubrutinib and subsequent AE reporting, given the practice change was initiated by a recommendation to providers from pharmacy with recent trial results.

In summary, to our knowledge, this is the largest cohort for real‐world analysis of zanubrutinib treatment patterns with the unique component of formulary change in an integrated community oncology practice. We found that zanubrutinib is effective and safe in patients with or without prior ibrutinib use. Zanubrutinib use had lower exposure‐adjusted incidence rates of cardiotoxicity than observed with ibrutinib use as seen in prior real‐world analyses, with decreased AE rates after BTKi switch with limitations as listed above [11]. Though not common in practice, few patients did tolerate rechallenge with zanubrutinib after prior progression on first‐generation BTKi. Additionally, in real‐world settings, discontinuation and dose reductions were most often due to low CTCAE grade AE. Further research is needed on the following: real‐world outcomes with longer follow‐up beyond that typically seen with randomized controlled trials, the effects of collective practice change from the patient perspective, the importance of lower‐grade AE reporting, and sequencing of multiple lines of BTKi therapy including acalabrutinib to zanubrutinib. This can help further characterize real‐world experiences of patients with chronic lifelong medications like BTKi in CLL/SLL.

## Author Contributions

All authors listed were contributing authors. M.L.K. was responsible for conceptualization, data curation, methodology, results interpretation, and writing the final report. B.H.C. contributed to data curation, results interpretation, and reviewing the final report. A.K.O. contributed to data curation, interpreting the results, and reviewing the final report. R.L. contributed to designing the protocol, results interpretation, and editing and reviewing the final report. L.L. contributed to results interpretation and editing and reviewing the final report. Z.Z. and S.Z. were responsible for data curation and analysis and reviewing the final report. L.C.S. contributed to data curation and analysis and editing and reviewing the final report. S.B. contributed to designing the protocol, results interpretation, and editing and reviewing the final report. M.M. contributed to results interpretation and editing and reviewing the final report. A.L. contributed to results interpretation and reviewing the final report. J.T. was responsible for designing the protocol, methodology, results interpretation, supervision of the study, and reviewing the final report.

## Ethics Statement

The study was approved by the Kaiser Permanente Institutional Review Board. Analyses were done in concordance with local committee recommendations respecting the Declaration of Helsinki.

## Conflicts of Interest

This study was funded by BeOne Medicines Ltd., San Carlos, CA, USA in partnership with Kaiser Permanente Northern California. Dr. Margaret Li Krackeler, Dr. Arthur K. Orchanian, Dr. Lisa Law, Dr. Alfredo Lopez, Zheng Zhu, and Shiyun Zhu have no competing financial interests to declare. Dr. Bryant H. Chee has an immediate family member employed at Pilant Therapeutics. Susan Buchanan is employed at BeOne Medicines as Medical Science Director and owns their stock. Dr. Raymond Liu has received travel expenses from Pfizer and research funding via his institution from Genentech, AstraZeneca, Exact Sciences, Biotheranostics, and BeOne Medicines. Dr. Lori C. Sakoda owns stock in JNJ and has received research funding via her institution from the National Institutes of Health, California Tobacco‐Related Disease Research Program, and AstraZeneca. Dr. Marjan Massoudi is employed at BeOne Medicines as the Principal Director, Medical Value & Outcomes Research Director, and owns their stock. Dr. Jahan Tavakoli has received research funding from BeOne Medicines.

## Supporting information


**Table S1:** cam471300‐sup‐0001‐TableS1.xlsx.

## Data Availability

The data that support the findings of this study are available from the corresponding author upon reasonable request.
